# Unlocking ovarian cancer heterogeneity: advancing immunotherapy through single-cell transcriptomics

**DOI:** 10.3389/fonc.2024.1388663

**Published:** 2024-05-30

**Authors:** Dharvind Balan, Nirmala Chandralega Kampan, Magdalena Plebanski, Nor Haslinda Abd Aziz

**Affiliations:** ^1^ Department of Obstetrics and Gynaecology, Faculty of Medicine, Universiti Kebangsaan Malaysia, Kuala Lumpur, Malaysia; ^2^ School of Health and Biomedical Sciences, RMIT University, Bundoora, VIC, Australia

**Keywords:** ovarian cancer, heterogeneity, single-cell sequencing, tumor-microenvironment, precision medicine

## Abstract

Ovarian cancer, a highly fatal gynecological cancer, warrants the need for understanding its heterogeneity. The disease’s prevalence and impact are underscored with statistics on mortality rates. Ovarian cancer is categorized into distinct morphological groups, each with its characteristics and prognosis. Despite standard treatments, survival rates remain low due to relapses and chemoresistance. Immune system involvement is evident in ovarian cancer’s progression, although the tumor employs immune evasion mechanisms. Immunotherapy, particularly immune checkpoint blockade therapy, is promising, but ovarian cancer’s heterogeneity limits its efficacy. Single-cell sequencing technology could be explored as a solution to dissect the heterogeneity within tumor-associated immune cell populations and tumor microenvironments. This cutting-edge technology has the potential to enhance diagnosis, prognosis, and personalized immunotherapy in ovarian cancer, reflecting its broader application in cancer research. The present review focuses on recent advancements and the challenges in applying single-cell transcriptomics to ovarian cancer.

## 1 Introduction

Ovarian cancer is one of the most common gynecological cancers that cause the highest mortality rate in women worldwide. In 2023, it was estimated around 13270 deaths from 19710 new cases which accounts for more than 67% of the mortality rate among women diagnosed with ovarian cancer (1). It is a type of heterogeneous group of malignancies with poor overall survival because of late-stage diagnosis and limited response toward the currently available treatment options. The World Health Organization (WHO) divides epithelial ovarian carcinomas (EOC) into several morphological groups based on cell type: serous carcinomas (SC), mucinous carcinomas (MC), endometrioid carcinomas (EC), clear-cell carcinomas (CCC), seromucinous carcinoma, Brenner tumors, mixed, and undifferentiated type (2). These subgroups differ in origin, appearance, molecular biology, and prognosis, yet are grouped (3). Cytoreductive surgery and platinum/taxane combined chemotherapy are the usual treatments whereby the response rate to first-line therapy is approximately 80–90%. Nevertheless, the majority of patients relapse and develop chemotherapy resistance, with a 5-year survival rate of 35% (4). Heterogeneity is a significant aspect of malignant tumors, explaining in part the lack of medical intervention.

**Figure 1:**
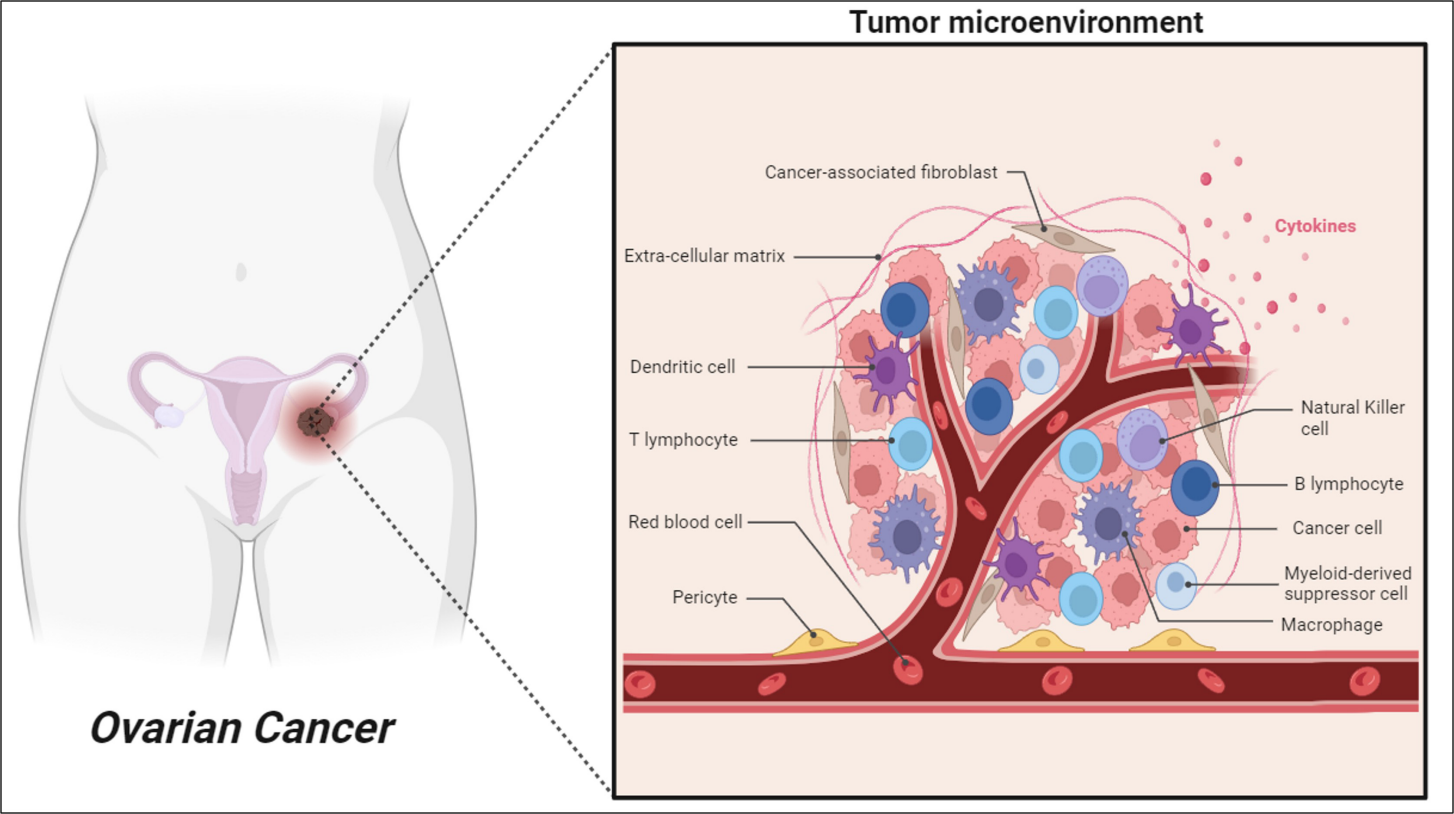
Schematic illustration of cellular diversity in ovarian tumor microenvironment.

Several clinical characteristics in ovarian cancer have confirmed a prominent role for the immune system in determining disease progression and outcomes. Ovarian cancer can induce an antitumor immune response by the host immune system found in different tumor microenvironments (TMEs) including blood, ascites and tumor tissue (5). Nevertheless, ovarian cancer presents multiple mechanisms of immune evasion, which consequently reduce the efficacy of immunotherapy to prevent the recurrence of disease and progression after surgery and chemotherapy (6). As illustrated in **Figure 1**, although there are presence of various anti-tumor immune cells such as B cells, T cells and Natural Killer cells (NK cells) in ovarian cancer TME, the co-existence of pro-tumor immune cells including dendritic cells (DCs), tumor-associated macrophages (TAMs), and myeloid-derived suppressor cells (MDSCs) effectively impairs the antitumor immunity and eventually allow tumor progression and metastasis (7, 8).

The intervention of immunotherapeutic approach including immune-checkpoint blockade therapy, adoptive T-cell therapies, and vaccine therapies has emerged as a promising alternative therapeutic method because of its enhanced specificity, long-term survival, and lower side effects for cancer treatment (8, 9). Among these, the immune checkpoint blockade therapy is the most promising approach in restoring anti-tumor immunity in many cancers but shows very minimal response rates in ovarian cancer due to its heterogeneity (9). Even though the biological and molecular heterogeneity of ovarian cancer has been investigated extensively, the heterogeneity of its TMEs remains unclear and warrants further studies. Thus, a new technological approach is needed to study the heterogeneity of immune cells to develop early diagnostic biomarkers, increase the treatment efficacy, reduce the disease burden, and improve the overall survival rate in ovarian cancer patients.

Single-cell sequencing (scRNA-seq) technology is a powerful tool advocated to investigate cellular heterogeneity by identifying genomic changes and different transcriptomic states at single-cell resolution. Conventional data obtained via bulk gene sequencing only represents an average gene expression over a dominant group of cells rather than the specific cell of interest or rare cells. Hence, the usage of scRNA-seq technology which can finely classify broad heterogeneity that exists within the tumor-associated immune cell populations and its TMEs will be ideal. Detailed investigation of the immune cell populations in ovarian cancer at a single cell level using scRNA-seq technology may also aid in the diagnosis of cancer as well as in the prognosis of immune-focused therapy (10). Single cell sequencing technology has been applied in the field of cancer providing favorable conditions for developing new tumor biomarkers and providing more accurate and individualized targeted immunotherapy for better clinical management of cancer patients (11). This article features the recent advances in single-cell transcriptome of tumor cells in ovarian cancer as well as discusses its application and future challenges.

## 2 Heterogeneity of ovarian cancer

Ovarian cancer is a complex disease with significant heterogeneity because of the existence of diverse subtypes or variations within a particular cancer type (3). Epithelial ovarian cancer is classified as a heterogeneous disease with several subtypes: high-grade serous, low-grade serous, clear cell, endometrioid, and mucinous ovarian cancer (12). Tumor heterogeneity appears to be quite high among subtypes and within a single tumor, posing a significant risk of treatment failure in ovarian cancer (3). Aside from the many subtypes of ovarian cancer, tumor heterogeneity, which can be classified into inter-tumoral and intra-tumoral heterogeneity, adds to the disease’s complexity. Inter-tumoral heterogeneity refers to the genotypic and phenotypic differences identified between numerous tumors of the same type in different individuals. For example, ovarian tumors in two distinct people might have quite varied profiles in terms of genetic alterations, cellular makeup, and responsiveness to therapy (13). Inter-tumoral ovarian cancer heterogeneity can be shown at multiple levels, including genetic, histological, and clinical features. On the other hand, intra-tumoral heterogeneity refers to genetic and biological differences that occur within a single patient caused by cancer cells, developing in response to specific environmental signals. Moreover, the presence of multiple cell types inside a single tumor mass, genetic differences between cancer cells, changes in the microenvironment, and the presence of a variety of cell types including immune cells and stromal cells can also result in intra-tumoral heterogeneity. Therefore, clonal diversity and variability of TME in intra-tumoral heterogeneity play an important role in metastasis, invasion, tumor growth, recurrence, and treatment resistance in ovarian cancer (14).

### 2.1 Genetic heterogeneity

Patients with ovarian cancer may have distinct genetic abnormalities. Mutations in tumor suppressor genes (such as BRCA1/2, PTEN, and TP53) and oncogenes (such as PIK3CA) are associated with a high risk of developing ovarian cancer (15, 16). Nevertheless, not all ovarian tumors have the same genetic changes, and the types and frequency of these alterations might vary. Numerous studies have shown that TP53 is mutated in 50% or more of high-grade serous carcinomas, whereas KRAS or BRAF activating mutations are prevalent in more than half of low-grade serous carcinomas (17, 18). Besides this, mutations in oncogenes, such as BRCA1/2, PTEN, and PIK3CA, have also been described in ovarian serous carcinomas, however, the frequency of mutation is generally modest (<10%) (17).

### 2.2 Histological heterogeneity

Histological heterogeneity in ovarian cancer refers to variances in the microscopic appearance of tumor cells across different patients. Ovarian cancer encompasses a spectrum of histological subtypes, each with distinct features and clinical outcomes, affecting prognosis and treatment effectiveness. The reliability of pathologists in accurately identifying these subtypes using current criteria ranges from high to moderate (19). Notably, there is a considerable discrepancy in differentiating between endometroid and serous carcinomas, leading to a reclassification of 20–30% of tumors initially diagnosed as endometrioid carcinomas (EC) to high-grade serous ovarian carcinomas (HGSOC) (20). Using a panel of immunohistochemical markers that includes at least WT1, p53, napsin A (NAPSA), and progesterone receptor (PR) significantly enhances histotype classification agreement. Although WT1 expression is characteristic of serous carcinomas, this marker does not have 100% sensitivity and specificity. The expression of p53 allows us to differentiate between HGSOC and LGSOC (21). NAPSA has been demonstrated to be a very sensitive and specific CCC marker. Its expression, on the other hand, might be poor and localized in some tumors, and it can occasionally be found in other histological types (22). Vimentin may aid in distinguishing PR-negative EOC with significant mucinous differentiation, which is typically vimentin-positive, from MOC, which is vimentin-negative (23).

### 2.3 Clinical prognostic determinants

Variations in clinical outcomes for ovarian cancer are influenced by several established prognostic determinants. The stage of the disease at diagnosis, the size of the primary tumor, and the extent of metastasis are critical markers that guide treatment decisions and influence patient prognosis. In addition to these tumor-centric factors, patient-specific determinants, such as age and overall health status, also play an essential role in shaping the clinical course and survival outcomes (24). HGSOC has the worst prognosis across all sub-types; nevertheless, it is important to realize that distant-stage MC and CCC have equal or worse 10-year survival projections than distant-stage HGSOC (25). Moreover, older age is related to poorer survival, with a median overall survival of 18.7 months compared to 53.2 months when comparing >70 years against 50–69 years (26). Molecular markers as prognostic variables, of which a lack of homologous recombination in DNA repair has been clinically verified, specifically the status of BRCA1/2 mutation. BRCA2 carriers fare better than BRCA1 carriers, while BRCA1/2 carriers fare better than BRCA wild-type patients (27). Chemotherapy, radiation, and targeted treatments can put selective pressure on cancers, resulting in the formation of resistant clones. This evolution adds to tumor heterogeneity, as well as highlights the difference between primary and recurring diseases. Populations that were previously small may become substantial contributors to recurring malignancies following the removal of other phylogenies by therapy, as evidenced in matched pre- and post-therapy clinical samples (28). These prognostic markers underscore the need for personalized therapeutic strategies and highlight the importance of considering tumor biology, patient characteristics and treatment responses in the management of ovarian cancer.

### 2.4 Clonal diversity

Clonal diversity refers to the presence of many cancer cell types in a single tumor, each with a distinct set of genetic mutations or epigenetic changes that can influence tumor development, metastatic potential, and resistance to medicines (29). The populations of ovarian tumor cells acquire unique characteristics over time as alterations accumulate, resulting in spatially and temporally different clones. Differentiation from clonal evolution, the presence of cancer stem cells, and tumor microenvironmental effects can all contribute to the formation of distinct cell populations (13). A study on the degree of clonal expansion between tumors was determined using a high-resolution whole-genome copy number method and selected genome-wide sequencing was done by Schwarz and colleagues (28). The study analyzed 135 tumor samples from 17 women undergoing chemotherapy for HGSC. The evolutionary history of each patient was determined by calculating the minimum number of events needed to transform genomic profiles. Interestingly, wide differences in clonal expansions between patients and within samples of the same tumor were observed but only a minor change was detected after treatment. Besides, Hoogstraat and team investigated the mechanism of treatment resistance driven by clonal expansion on 27 samples collected from primary and metastatic sites of 3 treatment naive HGSOC patients. The analysis of whole-genome mate-pair sequencing, topographic mapping of somatic break points, and transcriptional profiling revealed genomic alterations influence intratumor gene expression. Moreover, the study also unveiled the activation of different pathways in metastatic and primary tumors in the same patient, implying that tumor lesions at different sites appear to evolve independently and adapt to the environment (30).

### 2.5 Variability in tumor microenvironment

The tumor microenvironment (TME) consists of non-cancerous cells, including immune cells, fibroblasts, endothelial cells, and the extracellular matrix, which interact with tumor cells. Cancer cells communicate with these cells, exchanging chemical signals and establishing a niche where they may thrive in new environments. Variations in the TME composition can influence tumor progression, immune evasion, and response to treatment. Among the immune cells, CD8+ cells are more prevalent in HGSC than in other subtypes, with higher CD4+ cells and MHC-I expression in recurring cancers (31). In addition, the presence of immune cells will also differ by tumor location within a particular patient, whereby the degree of immune cell infiltration into a tumor and the clonal heterogeneity of that tumor are often inversely correlated (32). In addition to immune cells, local cell types such as cancer-associated fibroblasts (CAFs) also interact extensively with tumor cells. The presence of CAFs linked with metastatic tumor burden in patient samples, and it also facilitates invasion of ovarian cancer (33). The number of CAFs within the metastatic niche in the omentum expands along with the ovarian tumor growth. Zhang et al. also found that some omental samples contained CAFs but no cancer cells therefore suggesting CAFs promote angiogenesis and may work to prepare a niche before the emergence of ovaria cancer cells (33). As a result, in addition to circumstances influencing changes in tumor clones, the tumor itself may affect its surroundings. Moreover, ascites is a common feature of ovarian cancer, where tumor cells are exposed to a unique fluid environment within the peritoneal cavity. The tumor cells in this fluid are diverse, and they are exposed to a mix of pro- and anti-tumor signals from a wide range of cell types, such as CAFs, endothelial and mesothelial cells, immune cells, and other tumor cells. The content and proportion of soluble factors will alter as the disease advances, but they may include interleukins 6 and 8, which activate AKT and promote survival signaling in circulating tumor cells (34). On the other hand, tumor cells in ascites are exposed to exosomes from other tumor cells, which include proteins such as CD24 and the pro-apoptotic proteins Fas ligand and TRAIL (35). Therefore, understanding TME variability is crucial for designing therapies that target not just the tumor cells but also the supportive TME components.

Researchers and clinicians can gain a better understanding of ovarian cancer’s complexity by categorizing genetic variation and tumor heterogeneity with these categories. This comprehensive categorization facilitates the development of personalized treatment regimens, improves prognostic evaluations, and enhances patient-specific therapeutic outcomes.

## 3 Current treatments for ovarian cancer

The treatment techniques for various types of cancer are determined by their pathological phases. Early detection will aid in the development of promising and effective treatment alternatives. Ovarian cancer treatment typically involves a combination of surgery, chemotherapy, and, in some cases, targeted therapies. The specific treatment approach depends on the stage of the disease, histological subtype, genetic factors, and the patient’s overall health (36).

### 3.1 Debulking surgery

Surgery is a primary treatment for ovarian cancer. The goal is to remove as much of the tumor as possible in a procedure called debulking or cytoreductive surgery. The extent of surgery depends on factors such as tumor size, location, and spread. Surgery and systemic treatment are frequently used in tandem and are preceded by imaging procedures and a detailed pathologic report. Cytoreduction surgery is performed either at the time of the main diagnosis or after neoadjuvant chemotherapy (interval debulking surgery) (37). The outcome of surgery (whether complete or incomplete with no macroscopic remaining tumor) is critical for therapy planning. A pooled analysis showed the overall survival was 70, 40, and 30 months, respectively when complete cytoreduction, 0.1–1 cm residual disease, and >1 cm residual disease were attained during debulking surgery (38). Surgical options may include a hysterectomy (removal of the uterus), bilateral salpingo-oophorectomy (removal of both ovaries and fallopian tubes), and removal of any other affected tissues or lymph nodes (39).

### 3.2 Chemotherapy

Chemotherapy is commonly used in ovarian cancer treatment with the use of anti-cancer drugs that kill or inhibit the growth of cancer cells. The most frequently used chemotherapy regimen is a combination of platinum-based drugs (carboplatin) and a taxane drug (paclitaxel) (40). Chemotherapy can be given before surgery (neoadjuvant chemotherapy) to shrink the tumor or after surgery (adjuvant chemotherapy) to destroy any remaining cancer cells (41). Chemotherapeutic drugs can be given intravenously (IV), intraperitoneally (IP), or in a mix of the two. IP/IV chemotherapeutic agent delivery is the preferred form of administration of drugs for individuals with cytoreduced disease. Chemotherapeutic drugs are most effective when taken via IP route, which has some pharmacokinetic advantages such as increased IP concentration of the medication, a longer half-life of the drug in the abdominal cavity, and prolonged systemic exposure (42). An earlier study found that intraperitoneal chemotherapy following complete cytoreduction in stage III ovarian cancer was related to a lower mortality rate (43). Nevertheless, the use of high-dose chemotherapeutic agents will result in complications due to side effects and may result in the treatment plan being terminated. Because ovarian cancer cells undergo molecular-level alterations over time, they may develop resistance to treatment (36).

### 3.3 Targeted therapies

Targeted therapies are drugs that specifically target molecular alterations or pathways involved in cancer growth and progression and are also adopted as an alternative treatment modality in ovarian cancer management. For example, PARP inhibitors (such as olaparib, niraparib, and rucaparib) are used in patients with BRCA mutations, as these drugs exploit the DNA repair deficiencies in the cancer cells (44). Because these medications are taken orally and for lengthy periods without interruption, various adverse effects such as nausea, asthenia, and neutropenia have been reported (45). Intravenous bevacizumab, which targets angiogenesis, was the first biological agent approved for the treatment of ovarian cancer. Bevacizumab, a monoclonal antibody targeting vascular endothelial growth factor (VEGF), may also be used in combination with chemotherapy in certain cases (46).

### 3.4 Immunotherapy

Immunotherapy is quickly becoming the standard of care in a variety of human cancers. It aims to enhance the body’s immune system to recognize and attack cancer cells. Despite encouraging results in other malignant tumors, the use of single-agent antibodies inhibiting the CTLA-4, PD-1, or PD-L1 axis yielded only modest results in ovarian cancer, with median response rates of 10–15% and disease control observed in less than half of the patients (47). Nonetheless, due to a lack of prognostic biomarkers, no immunotherapeutic treatment has received regulatory approval for ovarian cancer to date. Therefore, a contemporary approach to employing immune checkpoint inhibitors is to combine them with anti-angiogenic treatments and PARP inhibitors, which could reduce primary resistance and improve therapy outcomes (48, 49).

It is vital to remember that treatment strategies are tailored to the unique characteristics of each patient’s malignancy. To make informed decisions regarding their care, patients should examine their treatment options, potential side effects, and long-term considerations with their healthcare team.

## 4 Biomarkers and molecular pathways in the development of ovarian cancer

Ovarian cancer is a highly heterogeneous disease and therefore poses a big challenge to biomarker discovery. There are various histological subtypes of epithelial ovarian cancer including serous, endometrioid and clear-cell carcinoma whereby each is indicated by different molecular features and characteristics. As a result, detecting ovarian cancer in its early stages will require a panel of tumor markers. Several clinically relevant epithelial ovarian cancer biomarkers discovered to date have already been reviewed in the past and present (50, 51). The standard approach for ovarian cancer diagnosis focused on the level of serum tumor biomarker CA-125 (carbohydrate antigen 125), which is raised in the serum of most ovarian cancer patients. Besides ovarian cancer, CA-125 is also highly expressed in other malignant and non-malignant conditions, therefore, limiting its ability to distinguish benign and malignant ovarian masses (52, 53). The lack of sensitivity and specificity of CA-125 to significantly detect the development of ovarian cancer at an early stage paves the way for the discovery of immunological biomarkers that have received special attention in the past years to study the likelihood of improving early cancer detection as well as to improve the survival rate among asymptomatic women.

Several reports over the decades have highlighted that ovarian cancer patients have high levels of serum cytokines leading to speculation that the possibility of the immune response toward ovarian cancer may have both diagnostic and prognostic value. Nevertheless, the use of individual cytokines is only limited to diagnostic tools due to concerns about their specificity and sensitivity. Therefore, measuring of variety of cytokines, especially in a multiplex manner could improve the diagnostic efficiency in ovarian cancer. A previous study demonstrated a combination of plasma IL-6 and IL-8, with two inflammatory markers, CRP and serum amyloid A with CA-125 levels enhanced the diagnostic efficiency compared to CA-125 alone (54). In another study, the combination of CA-125, IL-6, epidermal growth factor, vascular endothelial growth factor, and IL-8 was exceptional in distinguishing early-stage ovarian cancer from healthy controls with better sensitivity and specificity (55). Moreover, the biomarker panel of (CA-125, G-CSF, IL-6, vascular endothelial growth factor and epidermal growth factor) accurately distinguished benign masses from cancer with sensitivity and specificity of 84% and 76% respectively. These results collectively reveal the potential role of immune factors to be a useful biomarker panel for the early detection and diagnosis of ovarian cancer.

Natural immune responses to ovarian cancer have a crucial impact on the clinical outcome of the disease. Ovarian cancer can stimulate spontaneous anti-tumor immune responses whereby a significant number of tumor-infiltrating lymphocytes have been found in ovarian cancer tissues (56). T-cell infiltration into ovarian masses was associated with improved survival. A previous study reported the five-year survival rate is improved among patients with CD3^+^ T cells within their tumor compared to patients without infiltrating T cells after debulking and platinum-based therapy (57). Moreover, elevated numbers of intraepithelial CD8 cytotoxic T lymphocytes (CTL) also lead to improved survival as compared to those without intraepithelial CTLs (58). Nevertheless, the poor outcome of ovarian cancer is also contributed by immune cells that are unable to control tumor growth as a result of the recruitment of suppressive immune cells such as Tregs, or NK cells which fail to recognize tumor antigens (56). The role of tumor infiltrating immune cells is directly influenced by the level of circulating cytokines in the tumor microenvironment. Therefore, dissecting the heterogeneity of immune cells and the expression level of genes responsible for producing soluble cytokines at single-cell resolution can eventually determine the presence of immune cells and the cytokine levels in the ovarian cancer patient may help to predict the proper clinical outcome.

## 5 Single-cell sequencing technology

The conventional method of defining the cellular diversity both in tumor biopsies and TME respectively is using immunohistochemical (IHC) staining and flow cytometry. However, this is only limited to classifying the cell types based on specific cell surface markers, thus failing to dissect the intra-cellular variation. Moreover, the use of bulk RNA profiling to reveal the transcriptional state within the tumor and its TMEs has also failed to identify the respective contribution of each cell subset because this traditional method averages gene expression from highly distinct cell populations (59). Nevertheless, these limitations could be addressed by using scRNA-seq technology that combines the application of IHC, flow cytometry and RNA profiling in a single platform to investigate tumor composition, revealing cellular diversity and gene regulatory networks at a single-cell resolution (60–62).

**Figure 2:**
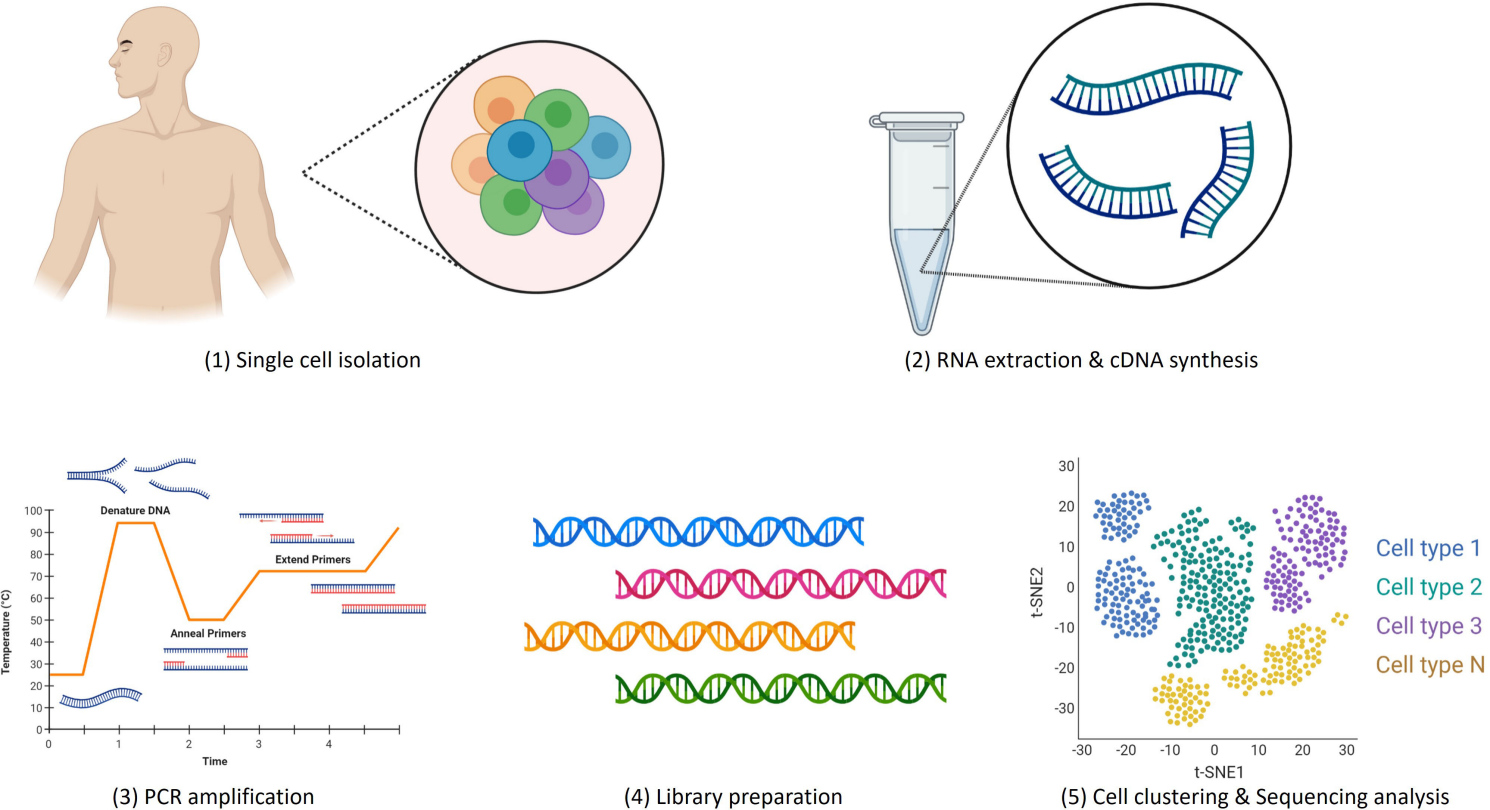
Single cell sequencing workflow.

Advancements in single-cell isolation, DNA sequencing, cDNA library preparation and bioinformatic analytical tools have led to remarkable progress in the development of scRNA-seq platforms in recent years. There are five crucial steps involved in scRNA-seq experiments: (1) single cell isolation (2), RNA extraction and cDNA conversion, (3) PCR amplification, (4) sequencing library preparation and (5) sequencing analysis as demonstrated in **Figure 2**.

The isolation of single cells is the most important step that determines the accuracy and quantity of DNA amplification. Single-cell isolation begins with cell selection by random seeding/dilution, laser microdissection (LCM), fluorescence-activated cell sorting (FACS), or microfluidic/microplate methods. Although FACS is mostly used to capture single cells, large numbers of cells for sequencing can be captured easily using microfluidic technology. This cutting-edge technique easily wraps single cells into an independent microdroplet that contains oligonucleotide primers and unique molecular identifiers (UMI) for cell identification. Nevertheless, there are several other improved technologies of FACS or microfluidics have been discovered over the past years to streamline and increase the throughput of single-cell isolation for transcriptomics, genomics, epigenomics, and proteomics studies. These new platforms also improved cell identification and library preparation for sequencing (63).

Single-cell RNA-seq is commonly used to profile the transcriptomes of individual cells whereby Droplet-based 10X Genomics Chromium and plate-based Switching Mechanism at the 5′ End of RNA Template sequencing (SMART-Seq) are two regularly used platforms (64). In 2012, SMART-Seq enabled the identification of full-length transcripts but as reported in 2013, SMART-Seq v2 excluded the purification step by replacing the last guanosine at the TSO 3′ end with locked nucleic acid (LNA) and improved protein thermal stability by using betaine has eventually increased the yield (65). SMART-Seq v2 is more sensitive and detects more genes inside a single cell, including low-abundance and alternatively spliced transcripts (66). However, SMART-Seq v4 improves efficiency in template swapping, resulting in faster cDNA synthesis and library development as well as providing increased sensitivity for low input and better consistency (67).

The high expense of single-cell DNA sequencing has hindered its adoption for high-dimensional analysis. Therefore, bulk sequencing is the most cost-effective approach, followed by targeted single-cell DNA sequencing to study specific mutations or variants of interest in cancer cells. TARGET-seq, which combines genomic and coding DNA genotyping, provides comprehensive coverage of important mutation hotspots and allows for sensitive investigation of known mutations inside individual cells (68).

Single-cell sequencing approaches, such as chromatin immunoprecipitation sequencing (ChIP-seq) and transposase-accessible chromatin assays using sequencing (ATAC-seq), can also identify epigenetic dynamics (69). Researchers used single-cell Cleavage Under Targets and Tagmentation (CUT&Tag) technology to analyze histone modifications in promoters, enhancers, and gene bodies, as well as regulatory interactions and chromatin occupancy within single cells. This technology provides high sensitivity and throughput (70). Moreover, *in situ* genome sequencing (IGS) uses DNA library creation, *in situ* sequencing, amplicon dissociation, PCR, and *ex situ* sequencing to pinpoint the precise location of a particular DNA sequence. As a result, it gives an important chance to address complex biological concerns, such as the links between genomic architecture and disease (71). These new approaches can identify the functions of transcriptomics and genomic architecture, as well as relationships between function, anatomy, transcription, and cell types in cancer and progression.

Deciphering the expressed proteome at the single-cell level is of tremendous interest as proteins are the primary functional machinery of cells. Mass cytometry by time of flight (CyTOF), which uses metal isotope-labelled antibodies and signal molecules to label cells, can identify 100 distinct proteins in a single cell, allowing for detailed quantitative proteomics sequencing at a single-cell level. On the other hand, Imaging mass cytometry (IMC) which was created from immunohistochemistry with metal-labeled antibodies, may assess up to 40 protein markers and their spatial architecture and relationships, providing information not available by standard tissue lysis of single cells (72). More importantly, IMC can be done on paraffin-embedded tissue slices, allowing for retrospective analysis of patient cohorts with known outcomes that can lead to tailored treatment.

## 6 Ovarian cancer at single-cell resolution

Single-cell RNA sequencing provides an unbiased and comprehensive method for examining the cellular heterogeneity and diversity within complex biological systems, such as the immune system. By analyzing the transcriptomes of individual cells, scRNA-seq enables the identification of distinct cell types and states without the preconceived biases inherent in bulk sequencing methods (73). Single-cell gene expression profiles using scRNA-seq technologies in immunological studies has transformed our understanding of cellular development and differentiation at the molecular level and the role of immune cells in health and disease. Investigation of the whole transcriptome at single-cell resolution by the scRNA-seq platform enables the discovery of novel regulators of immune cell differentiation and the deciphering of the heterogeneity in the immune system (74, 75). To date, most transcriptomic studies using scRNA-seq technology in ovarian cancer have aimed mainly at cancer cells while only a handful of studies have investigated the heterogeneity of TME which contains various immune cell types that are also crucial for patient stratification, targeted treatment planning, and predict prognostic outcomes. **Table 1** summarizes recent findings of scRNA-seq in ovarian cancer.

**Table 1:** Summary of recent findings of scRNA-seq in Ovarian cancer.

Source	Sample Type	Sample Size	Treatment	Findings	Platform	References
Ovarian tumor biopsy	Primary HGSOC tumor biopsy	N=1 92 single cells from tumor tissues	Pre-chemotherapy	1. Identification of two major cell subsets characterized based on epithelial (proliferative genes, and genes related to oxidative phosphorylation and MYC activity) and stromal (extracellular matrix genes and genes associated with epithelial-to-mesenchymal transition) gene expression patterns. 2. Neither of these groups displayed gene expression patterns associated with chemoresistance based on three independent studies.	Fluidigm C1 chip, Illumina HiSeq2500	(76)
	Primary HGSTOC tumor biopsy	N=7 18,403 single cells from 7 tumor tissues	Pre-chemotherapy	1. Detection of 11 cancer cell and 32 stromal cell subtypes deriving from both the primary ovarian tumor and its metastatic peritoneal or omental lesions. 2. Discovery of 43 new potential targets for therapy and 6 cellular phenotypes of prognostic significance. 3. Cells group of 21 clusters representing 8 major cell types based on canonical marker gene expression across these clusters including epithelial cancer cells, myeloid cells, dendritic cells, T cells, B cells, fibroblasts, endothelial cells and ovarian stromal cells.	Chromium Single Cell 3’ Library, Gel Bead & Multiplex kit and chip kit (10X Genomics), Illumina HiSeq 4000	(77)
	Metastatic HGSOC/malignant tumor biopsy	N=6 9,885 single cells from omental tumor samples of 6 patients	Pre-chemotherapy = 2/6 Post-chemotherapy = 4/6	1. Identification of unique sub-populations of CD274^+^ and IRF8^+^ macrophages, CD4^+^GNLY^+^ T cells, plasmablasts and plasma B cells. 2. Transcriptional analysis of immune cells stratifies our patient samples into 2 groups: (1) high T cell infiltration and (2) low T cell infiltration. 3. Plasmablast and plasma B cell clusters, and NR1H2^+^IRF8^+^ and CD274^+^ macrophage clusters, suggesting an anti-tumor response in the high Tinf group.	Drop-seq microfluidic, Illumina’s NextSeq 500	(59)
	Primary HGSOC tumor biopsy	N=12 5000 cells from intact tumor tissues	Post-chemotherapy	1. Discovery of the presence of more T-cells, B-cells and natural killer cells by *in-situ* single-cell RNA sequencing technique that promotes better progression-free survival and overall survival in excellent neoadjuvant chemotherapy responders. 2. Identification of highly expressed several DNA repair genes (ATR, ARID2, and ARID1B) in poor responders compared to bulk sequencing. 3. Discovery of downregulation in immune cell activation, adaptive immune response, and regulation of innate response pathways in poor responders.	10X Genomics, Illumina’s NovaSeq 6000	(78)
Ovarian tumor biopsy	Primary HGSOC tumor biopsy	N=12 59,324 cells from tumor & nonmalignant tissues	Pre-chemotherapy	1. A series of epithelial-to-mesenchymal transition EMT-associated gene signatures (NOTCH1, SNAI2, TGFBR1, and WNT11) distinguished tumor cells and could be applied to predict poor patient outcomes. 2. The dominant CAFs in HGSOC tumors were mCAF expressing -SMA, vimentin, COL3A, COL10A, and MMP11, which induces EMT properties in ovarian cancer cells in the coculture system. 3. In early-stage cancers, certain immune cell subsets such as C7-APOBEC3A M1 macrophages, CD8^+^ T_RM_, and T_EX_ cells were selectively enriched.	10X Genomics, Illumina sequencer	(79)
	Metastatic omental HGSOC tumor biopsy	N=3 2571 cells from omental tumor tissues	Pre-chemotherapy	1. Identified over 150000 cell-type-specific isoforms using short-read and long-read scRNA-seq. 2. On average, 20% of protein-coding gene expression was non-coding, resulting in an overestimation of protein expression. 3. In omental metastases, mesothelial cells transition into CAFs is mainly through the TGF-β/miR-29/Collagen axis.	10X Genomics, Illumina NovaSeq platform	(80)
	Ovarian cancer tumor biopsy	N=2 5000–10000 cells	N/A	1. Uncovered a cell subcluster C4, which is strongly related to metastasis and a poor prognosis in ovarian cancer. 2. The C4 subcluster exhibited an EMT and angiogenesis signature, with RAB13 serving as a significant marker. 3. Downregulation of RAB13 reduced OC cell migration and invasion.	10 × Genomics Chromium platform Illumina Novaseq 6000	(81)
Tumor microenvironment	Ascites from advanced HGSOC	N=11 11000 cells from ascites	Treatment naive, pre- and post-chemotherapy	1. The composition and functional programming of ascites cells vary significantly between patients. 2. The mesenchymal subtypes are immunoreactive and with prognostic significance. 3. The JAK/STAT pathway, which was expressed in both malignant cells and cancer-associated fibroblasts, was found to have a powerful anti-tumor effect.	10X Genomics, Illumina NextSeq 500	(24)
	Ascites from advanced HGSOC	N=14 53499 cells from ascites	Post- chemotherapy	1. Memory T cells are concentrated in ascites as a reservoir for tumor-infiltrating depleted CD8^+^ T cells and T helper 1-like cells. 2. Tumor-enriched macrophages favored monocyte-derived ontogeny, while ascites macrophages favored embryonic ontogeny. 3. As prognostic biomarkers for platinum-based chemotherapy response, MAIT and dendritic cells in malignant ascites, as well as two endothelial subsets in primary tumors, have been identified.	10X Genomics, Illumina’s NovaSeq 6000	(82)

ARID1B, AT-rich interactive domain 1B; ARID2, AT-rich interactive domain 2; ATR, Ataxia telangiectasia and Rad3-related protein; CAF, Cancer-associated fibroblast; CD274, Programmed death-ligand 1; CD4, Cluster of differentiation 4; COL3A, Type III collagen; COL10A, Type X collagen; EMT, Epithelial-to-mesenchymal transition; GNLY, Granulysin; HGSOC, High-grade serous ovarian cancer; HGSTOC, High-grade serous tubo-ovarian cancer; IRF8, Interferon regulatory factor 8; LYPD6, LY6/PLAUR Domain Containing 6; MAIT, mucosal‐associated invariant T cells; mCAF, Matrix cancer-associated fibroblast; MMP11, Matrix Metallopeptidase 11; NOTCH1, Neurogenic locus notch homolog protein 1; SLAMF7, SLAM Family Member 7; SMA, Smooth muscle actin; SNAI2, Zinc finger protein SNAI2; TAMs, Tumor-associated macrophages; TBX2-AS1, TBX2 Antisense RNA 1; T_EX_, Exhausted T cells; TGF-β, Transforming growth factor-beta; TGFBR1, Transforming growth factor-beta receptor type 1; TME, Tumor microenvironment; T_RM_, Tissue resident memory T cells; WNT11, Wnt Family Member 11; N/A, not available.

In a study conducted by Winterhoff, B. J., and colleagues, two primary groups of cells, epithelial and stromal gene expression patterns have been found in high-grade serous ovarian cancer (HGSOC) patient tumor cells (76). The epithelial group was distinguished by proliferative genes, such as those involved in oxidative phosphorylation and MYC activity, whereas the stromal group was distinguished by increased expression of extracellular matrix (ECM) genes and those involved in epithelial-to-mesenchymal transition (EMT). Although neither group showed a signature that corresponded to previously described chemo-resistant gene signatures, numerous cells, primarily in the stromal subgroup, displayed markers associated with cancer stem cells, suggesting they might not accurately indicate resistance. Moreover, the patient in focus, showing no recurrence after surgery, had single cells without a chemo-resistant gene pattern. On the other hand, this study’s patient is classified as mesenchymal at the bulk sample level but shows a mix of mesenchymal and proliferative subtypes at the single-cell level, hinting at multiple molecular subgroups within tumors. This differential outcome at bulk versus single cell level indicates the potential use of scRNA analysis to categorize cancer cell subpopulations affecting clinical outcomes and chemo responses in ovarian cancer.

Siel Olbrecht and co-researchers found 11 cancer and 32 stromal cell morphologies originating from primary ovarian tumors and their metastatic lesions in high-grade serous tubo-ovarian cancer (HGSTOC) patients by using scRNA-seq (77). This study emphasizes patient-specific cancer cell clusters based on genetic variations in tumors. Some cancer cell subclusters display similarities with previously identified oncogenic pathways, potentially responsible for HGSTOC development and maintenance. The number of myofibroblasts, TGF-driven cancer-associated fibroblasts, mesothelial cells, and lymphatic endothelial cells indicated poor prognosis, whereas plasma cells suggested better outcomes in patients. Furthermore, they also discovered a distinct cell-like transcriptome signature in cancer cells, which was associated with worse overall survival in HGSTOC patients. The phenotypes of stromal cells varied greatly amongst molecular subgroups. The mesenchymal, immunoreactive, and differentiated signatures, for example, were distinguished by distinct fibroblast, immune cell, and myofibroblast/mesothelial cell morphologies suggesting the benefit of scRNA-seq to identify stromal cell characteristics that predict overall survival in patients with HGSTOC. However, they have also highlighted the need for more extensive analysis involving a larger number of patients and various sampling sites to validate the patient’s stratifying strategy based on these phenotype-specific marker genes that could be a potential technique for predicting prognosis or responsiveness to therapy in ovarian cancer.

A recent study published in 2023 performed high-throughput long-read scRNA-seq to capture cell-type-specific genomic and transcriptomic alterations on tumor samples collected from three HGSOC patients presenting with omental metastasis (80). Dondi and colleagues used both short-read and long-read scRNA-seq in 2571 individual cells to generate the deepest dataset which enabled them to identify 150000 cell-type-specific isoforms of which 52,000 were not previously reported. An isoform-level investigation indicated that, on average, 20% of protein-coding gene expression was noncoding, resulting in an overestimation of protein expression. The study discovered that in omental metastases, mesothelial cells change into CAFs via the TGF-β/miR-29/Collagen axis, based on differential isoform and polyadenylation site usage analysis of cells from metastatic TME and distant omental biopsies. They also observed genomic and transcriptomic dysregulations in the insulin-like growth factor (IGF) network in tumor cells. As a result, this study demonstrated that scRNA-seq may reliably capture genomic changes, including cancer- and patient-specific germline and somatic mutations in genes like TP53, as well as gene fusions, such as an IGF2BP2:TESPA1 fusion.

Another study on metastatic ovarian cancer utilized scRNA-seq to identify 9 primary cell types, including cancer, stromal, and immune cells, in 9,885 cells isolated from the omentum of 6 patients (59). The study identified distinct clusters of cancer epithelial cells expressing genes associated with metastasis and EMT, alongside the presence of cancer stem cell-like populations. Their transcriptional analysis of immune cells divides patient samples into two groups: (1) those with strong T cell infiltration (high T_inf_) and (2) those with low T_inf_. The high T_inf_ group is enriched in TOX-expressing resident memory CD8^+^ T (CD8^+^ T_rm_) and granulysin-expressing CD4^+^ T cell clusters potentially impacting cancer therapy, especially in the context of immune checkpoint blockade. Concurrently, they also discovered distinct plasmablast and plasma B cell clusters, as well as NR1H2^+^IRF8^+^ and CD274^+^ macrophage clusters, indicating an anti-tumor response in the high T_inf_ group. These findings suggest a potential link between these cell populations and their role in response to immune checkpoint inhibitors, emphasizing the need for mechanistic studies to enhance patient responses to these therapies. Nonetheless, the study notes limitations due to low cell counts in certain cell types, restricting further analysis. It also highlights the absence of mesothelial cells in their metastatic dataset from the omentum, contrary to findings in benign ovarian tumors in previous studies (10).

Although many studies have explored the mechanisms and treatment of ovarian cancer at single cell level, the primary focus was given to the commonalities among multiple patients limited by the sample size and the high inter-tumor heterogeneity of ovarian cancer. Nevertheless, a study by Guo et al. successfully constructed a cell atlas containing normal epithelium, primary carcinoma, and metastatic carcinoma by integrating single-cell sequence data from 12 patients (81). In this study, the developmental trajectory of cancer cells during metastasis was characterized using pseudo-time trajectory analysis at various phases, and a cell subcluster with commonality in patients and close association with metastasis was found. This subcluster has immune escape and pro-mesenchymal growth features, as indicated by cell-cell communication studies. Moreover, an overexpressed gene RAB13, that had not before been reported in ovarian cancer was found to increase cell migration and invasion *in vitro*. Further exploration of the associations between RAB13 expression levels and clinical phenotypes as investigated using TCGA datasets shows RAB13 to be strongly related to a poorer prognosis and tumor progression. This suggests that RAB13 could be further explored to have a deeper understanding of the mechanisms for ovarian cancer metastasis and as a potential drug target.

Interestingly, both bulk and scRNA-seq do not give a complete evaluation of tissue spatial diversity in cancer samples, and existing *in situ* methods (multiplex immunohistochemistry and imaging mass cytometry) only allow for restricted study of a small number of targets (83). Stur, E., et al. is the first scientific group to use intact tumor tissue to conduct a complete approach to spatial transcriptomics of HGSOC (78). They chose a small group of patients with highly annotated HGSOC, divided them into two groups based on their response to neoadjuvant chemotherapy (poor or excellent), and examined pre-treatment tumor tissue specimens. The team discovered significant changes in tumor composition between poor and excellent treatment responders, which were related to cell cluster architecture and location. The significance of the stromal component influencing chemotherapy response and the identification of diverse cell clusters in different tissue types, particularly concerning the EMT pathway. The study reveals variations in EMT pathway activity within and among tumor tissues, suggesting specific cell populations may contribute to therapy resistance. It emphasizes the importance of physical cell cluster connections within the TME, such as the mesenchymal cluster’s interactions possibly sustaining resistance and immune cell clusters enhancing chemotherapy’s cytotoxic effects. This detailed analysis of tumor tissue from poor and good responders with high-grade serous ovarian cancer revealed that spatial interactions between cell clusters may impact chemo-responsiveness more than cluster composition alone.

In addition, a recent study produced a high-resolution representation of the cellular contact network in early- or late-stage HGSOC tumors compared to nonmalignant ovarian tissues (79). J Xu and co-scientists unveiled the distinct TME elements of HGSOC and tumor cell traits linked with tumor phases using scRNA-seq techniques. They identified 38 genes of the EMT programme differentially expressed in HGSOC tumor cells when compared to nonmalignant ovarian cells. Among these, expression levels of EMT markers consisting of NOTCH1, SNAI2, TGFBR1, and WNT11 were associated with poor survival in at least three cohorts similar to worse patient overall survival in all The Cancer Genome Atlas and Gene Expression Omnibus serous ovarian cancer cohorts. Moreover, they also found a highly immunosuppressive context in which most invading tumor specific CD8^+^ T lymphocytes become exhausted and effector function is substantially compromised in HGSOC patients. Although several T-cell coinhibitory receptors have been identified including TIGIT, CTLA4, HAVCR2, LAG3, PDCD1, and SIRPA, TIGIT was the most abundant coinhibitory receptor on CD8^+^ T exhausted cells. Interestingly, TIGIT inhibition hampered tumor development in mouse models of patient-derived ovarian cancer and greatly reduced the frequency of TIGIT^+^-CD8^+^ T cells in tumors. On the other hand, the ability of macrophages to recruit immune cells was steadily diminished, whilst the effects of growth factor release were dramatically boosted as the phases progressed, showing that malignant transformation of macrophages occurred during this process. In this study, a distinct subpopulation of APOBEC3A M1 macrophages were mostly in early stage 1, had increased chemokine production, and were linked with better survival outcomes.

Ascites, a cluster of cell types, are found in one-third of ovarian cancer patients at the stage of diagnosis and are common in individuals with chemotherapy-resistant disease (84). The accumulation of malignant abdominal fluid is a common complication in women with advanced HGSOC, and it is related to resistance to drugs and an uncertain prognosis (85). In a study published in 2020, the researchers performed sc-RNA sequencing to analyze 11,000 cells from 22 ascites specimens from 11 individuals with HGSOC to define the HGSOC ascites ecosystem (24). The study of malignant ascites from patients with advanced HGSOC using scRNA-seq revealed considerable differences in cellular states and programs between malignant and non-malignant cells. Cancer-associated fibroblasts (CAFs) (CD14, AIF1, CSF1R and CD163 positive cells) are a subgroup with immunomodulatory programs in which inflammatory CAFs produce IL-6 and other cytokines and may promote tumor development and treatment resistance. Moreover, macrophage diversity was predominantly driven by two gene programs: one involving major histocompatibility complex (MHC) class II, interferon-receptor 1, and M1-associated genes, and the other by complement factors, demonstrating that an equilibrium of these phenotypes existed within the ascites environment of patients getting the platinum therapy. Malignant cells that express the MHC class II program may have more tumor-infiltrating lymphocytes, a better prognosis, and a stronger immunotherapy response. The ascites ecosystem may balance cancer progression and medication responses, and changing this equilibrium therapeutically might help alter the drug-resistant setting. Furthermore, the differentiated subtype program is robustly expressed by most cancer cells across patients, whereas the proliferative subtype is expressed by a minority. The mesenchymal and immunoreactive subtype programs are not expressed by cancer cells, but rather by CAFs and macrophages, which represent tumor makeup. CAFs can explain the majority, if not all, of the mesenchymal subtype, implying that cancer cells play an important role in tumor composition. However, future research should look at a bigger amount of patient samples to see if the programs revealed in one patient are generalizable. Single cell profiling of well-stratified clinical cohorts is also required to improve inter-patient comparisons, find convergent elements of tumor biology and drug resistance, and improve our understanding of HGSOC.

Another well-constructed study conducted by Zheng, X., et al. has outlined a complete landscape of ovarian cancer TME using scRNA-seq by analyzing the distinct cellular components of five tumor-related locations, including primary ovarian tumor, omentum metastasis, ascites, pelvic lymph node, and peripheral blood (82). The researchers used scRNA-seq to investigate the intricacy of TME as well as the relationships between the samples obtained from five different tissues in 14 patients with ovarian cancer who had variable sensitivity to platinum-based treatment. They found ascites-derived GZMK^+^ T_EM_, which resembles ‘pre-exhausted’ CD8^+^ T cells within tumors, might be a substantial source of tumor-infiltrating T_EX_ cells, implying that ascites-derived memory T cells may migrate into tumor locations and serve as a key cell pool for TILs. In addition, accelerating the movement of ascites derived GZMK^+^ T_EM_ cells into tumor locations might be a possible ovarian cancer treatment method. More research is needed, however, to completely understand the functional functions of these ascites T lymphocytes. On the other hand, conventional DCs demonstrated distinct ascites-enriched distribution patterns, suggesting that their presence in ascites might serve as a possible source of LAMP3^+^ DCs in tumor tissues. Macrophages of various origins and phenotypes coexisted inside the ovarian tumor and ascites, with tumor-enriched macrophages regulating immunity and ascites-enriched macrophages being more pro-inflammatory. Moreover, DES^+^ mesothelial cells in ascites and IL13RA1^+^ endothelial cells at the tumor site are two examples of stromal cell types that play essential roles in tumor growth. Ascites-enriched DES^+^ mesothelial cells may assist in remolding the ascites milieu by attracting T cells and macrophages via CXCL12-CXCR4. Lastly, IL13RA1^+^ endothelial cells with tip-like characteristics were found to be considerably abundant in platinum-resistant individuals indicating its influence on chemotherapy resistance. The findings shed light on the biological factors that contribute to the remodeling of the TME and identified specific cell subpopulations that could serve as potential predictive biomarkers for chemotherapy and prognostic markers for prolonged survival, while also discovering new therapeutic targets or methods for overcoming platinum resistance and immune suppression in ovarian cancer.

## 7 Potential clinical applications of scRNA-seq in ovarian cancer

Single-cell sequencing has emerged as a powerful tool in cancer research and has several clinical applications in ovarian cancer. By analyzing individual cells’ genetic and molecular characteristics, single-cell sequencing allows researchers and clinicians to gain valuable insights into tumor heterogeneity and potential treatment strategies (86). It shows tumor cell heterogeneity and monitors tumor progression, preventing additional cellular damage. Moreover, immune cell transcriptome analysis in tumor tissue can be utilized to classify immune cells, their immune escape mechanisms, and drug resistance mechanisms, as well as to design effective therapeutic targeted therapies in combination with immunotherapy in ovarian cancer (87). Here are some key potential clinical applications of scRNA-seq for the management of ovarian cancer.

### 7.1 Understanding cellular diversity

Ovarian cancer is known for its high degree of cellular diversity, with different cell populations within the same tumor displaying distinct genetic and molecular features (88). Single-cell sequencing enables the identification and characterization of various cell subpopulations, providing a comprehensive view of the tumor’s complexity (89). This may help researchers understand the composition of tumor tissues, identify rare cell types, and explore how different cell populations interact within the tumor microenvironment. Moreover, this information can be essential in designing personalized treatment plans that target specific cell populations driving tumor growth and resistance.

### 7.2 Predicting treatment response

Single-cell sequencing can identify genetic mutations and gene expression patterns associated with drug resistance or response to specific therapies. This sequencing approaches provide precise and reliable profiling of tumor subpopulations, revealing modest changes in therapy response. Deep transfer learning to predict drug sensitivity allows us to use not just past information derived from enormous bulk sequencing data, but also the diverse landscapes produced by single-cell sequencing techniques (90). By analyzing individual cells, researchers can identify subpopulations that may be responsible for treatment resistance. This information can aid clinicians in selecting the most effective treatment options for individual patients, improving treatment outcomes.

### 7.3 Biomarker discovery

Traditionally, bulk transcriptome signatures have been employed to identify prognostic biomarkers in cancer but have not yet demonstrated convincing clinical utility. It also lacks the resolution to capture important cell types in tumors and their complex microenvironment; therefore the true nature of epithelial cell diversity remains unknown (91). Single-cell sequencing enables the discovery of novel biomarkers that can aid in early diagnosis and prognosis of ovarian cancer. Researchers can develop more accurate and sensitive biomarkers for detecting ovarian cancer and monitoring its progression by identifying unique genetic and molecular signatures in specific cell populations.

### 7.4 Uncovering immune responses

The tumor microenvironment in ovarian cancer is influenced by immune cells that can either promote or inhibit tumor growth (7). In ovarian cancer, the tumor microenvironment is important in developing therapy resistance and disease progression because it provides cancer stem cell niches, promotes tumor cell metabolic reprogramming, reduces chemotherapeutic drug perfusion, and leads to an immunosuppressive environment (92). Single-cell sequencing can characterize immune cell populations and their interactions within the tumor cells precisely. This information can be used to develop immunotherapies or combination therapies that enhance the body’s immune response against ovarian cancer. Moreover, the treatment response rate of immunotherapeutic agents could also be retrieved by single-cell sequencing techniques.

### 7.5 Monitoring minimal residual disease

After surgery and chemotherapy, there may still be residual tumor cells that are not detectable by conventional methods. Patients with macroscopic residual disease (0.1–0.5 cm) outlive those with more than 0.5 to 1 cm (53 months) (38). Minimizing the occurrence of residual disease post-surgery and chemotherapy could improve the survival rate of ovarian cancer patients. Single-cell sequencing can provide more sensitive detection of minimal residual disease, helping clinicians assess treatment effectiveness and make informed decisions regarding further therapy. This would eventually lead to better management of ovarian cancer patients and reduce the chance of cancer relapse.

Overall, the clinical application of single-cell sequencing in ovarian cancer holds significant promise for advancing precision medicine approaches and improving patient outcomes by tailoring treatments to individual tumor characteristics. However, it is important to note that single-cell sequencing is still an evolving technology and further research, and validation are needed before its widespread clinical implementation.

## 8 Discussion

Ovarian cancer is a highly heterogeneous disease with various subtypes and variations, which poses challenges in its treatment. This heterogeneity can be observed at genetic, histological, and clinical levels. Ovarian cancer patients may exhibit different genetic abnormalities, with mutations in various genes associated with the risk of ovarian cancer. For instance, high-grade serous carcinomas often have TP53 mutations, while low-grade serous carcinomas frequently have KRAS or BRAF mutations. Other oncogenes like BRCA1/2, PTEN, and PIK3CA are also involved but less frequently. The microscopic appearance of ovarian cancer cells varies across different histological subtypes, leading to differences in clinical behaviors and treatment responses. Pathologists may have challenges in distinguishing between certain subtypes, such as endometrioid and serous carcinomas, which can lead to reclassification. Clinical aspects of ovarian cancer, including its presentation, progression, and response to treatment, can differ significantly. Factors like cancer stage, tumor size, and metastasis, as well as patient characteristics like age and overall health, influence the clinical outcome. Understanding ovarian cancer’s heterogeneity is crucial for tailoring personalized treatment approaches.

Treatment strategies for ovarian cancer are determined by factors such as the cancer’s stage, histological subtype, genetic factors, and the patient’s overall health. Surgery aims to remove as much of the tumor as possible whereby the extent of surgery depends on tumor characteristics. Complete cytoreduction during surgery is critical for therapy planning and can significantly impact survival. In addition, chemotherapy using platinum-based drugs such as carboplatin and taxane is commonly used in combination with ovarian cancer. Chemotherapy is often administered before surgery to shrink the tumor or after surgery to eliminate any remaining cancer cells. Nevertheless, targeted therapies which focus on specific molecular alterations or pathways involved in cancer growth have gained popularity among clinical researchers in recent times. The PARP inhibitors are used in patients with BRCA mutations, exploiting DNA repair deficiencies in cancer cells. Moreover, Bevacizumab, which targets angiogenesis, can also be used in combination with chemotherapy. Besides that, immunotherapy, which boosts the body’s immune system to recognize and attack cancer cells, is an emerging treatment option which warrants in-depth studies to be employed in ovarian cancer. This is because its effectiveness in ovarian cancer has been modest, and no immunotherapeutic treatment has received regulatory approval to date. Combining immunotherapy with anti-angiogenic treatments and PARP inhibitors is being explored to enhance therapy outcomes. In the ovarian cancer ecosystem, each patient’s treatment plan is customized based on their unique cancer characteristics, and patients need to discuss their options, potential side effects, and long-term considerations with their healthcare team. Early detection is always crucial for more promising and effective treatment options that need new technological interventions in cancer management.

Ovarian cancer is a highly heterogeneous disease, which makes biomarker discovery challenging. Different histological subtypes, such as serous, endometrioid, and clear-cell carcinoma, have distinct molecular features and characteristics. Detecting ovarian cancer in its early stages necessitates a panel of tumor markers. The conventional diagnostic approach has relied on the serum tumor biomarker CA-125, which is elevated in most ovarian cancer patients. However, CA-125 is also found in high levels in other benign and malignant conditions, limiting its ability to differentiate between benign and malignant ovarian masses. Due to the lack of sensitivity and specificity of CA-125, there has been a focus on the discovery of immunological biomarkers in recent years to improve early cancer detection and enhance the survival rate among asymptomatic women.

The presence of high levels of serum cytokines in ovarian cancer patients has sparked interest in the potential diagnostic and prognostic value of the immune response to ovarian cancer. However, individual cytokines have limitations in terms of specificity and sensitivity for diagnosis. Multiplex measurements of various cytokines need further advancement to improve diagnostic efficiency in ovarian cancer. Besides this, the natural immune responses to ovarian cancer play a critical role in the clinical outcome of the disease. The presence of tumor-infiltrating lymphocytes, particularly T cells and CD8 cytotoxic T lymphocytes (CTLs), has been associated with improved survival in ovarian cancer patients. However, the effectiveness of the immune response can be influenced by suppressive immune cells like Tregs and NK cells, as well as the levels of circulating cytokines in the tumor microenvironment. Therefore, dissecting the heterogeneity of immune cells, examining the expression of genes responsible for cytokine production at a single-cell resolution, and determining the presence of immune cells and cytokine levels in ovarian cancer patients may be valuable for predicting clinical outcomes in ovarian cancer.

Conventional techniques, such as IHC staining, flow cytometry, and bulk RNA profiling, have restrictions in dissecting both inter-cellular and intra-cellular heterogeneity of ovarian cancer. These techniques mainly rely on the expression of surface markers and result from averaging of gene expression from diverse cell populations. ScRNA-seq addresses these limitations by combining elements of IHC, flow cytometry, and RNA profiling within a single platform, allowing for the investigation of cellular composition, revealing cellular diversity, and uncovering gene regulatory networks at a single-cell resolution. Recent advancements in single-cell isolation, DNA sequencing, cDNA library preparation, and bioinformatics tools have significantly improved scRNA-seq platforms. Various methods, FACS, microfluidics, and laser microdissection, are used to isolate single cells, with microfluidic technology being particularly efficient for high-throughput single-cell capture. Fast-paced developments in FACS and microfluidics, further streamline single-cell isolation processes and enhance cell identification and library preparation for sequencing, thus improving our understanding of cellular heterogeneity in cancer.

Single-cell sequencing has emerged as a powerful tool in ovarian cancer research and offers several clinical applications. It provides valuable insights into tumor heterogeneity, immune responses, and treatment strategies. The use of scRNA-seq enables the discovery of novel biomarkers for early diagnosis and prognosis of ovarian cancer. It offers higher resolution to capture the complexity of cell types in tumors and their microenvironment, leading to the development of more accurate and sensitive biomarkers. Single-cell sequencing allows for the comprehensive identification and characterization of highly heterogeneous ovarian cancer subpopulations, helping researchers or clinicians understand the complexity of tumor tissues. This information is vital for designing personalized treatment plans targeting specific cell populations that drive tumor growth and resistance. Furthermore, scRNA-seq can identify genetic mutations and gene expression patterns associated with drug resistance or response to specific therapies. By analyzing individual cells, researchers can pinpoint subpopulations responsible for treatment resistance, guiding clinicians in selecting the most effective treatment options for individual patients. Ovarian cancer’s microenvironment is influenced by immune cells that can either promote or inhibit tumor growth. Single-cell sequencing precisely characterizes immune cell populations and their interactions, aiding in the development of immunotherapies and combination therapies to enhance the body’s immune response against ovarian cancer. In addition, this cutting-edge technology provides a more sensitive method for detecting minimal residual disease after surgery and chemotherapy. This helps clinicians assess treatment effectiveness and make informed decisions regarding further therapy, ultimately improving the management of ovarian cancer patients and reducing the risk of cancer relapse.

In the nutshell, single-cell sequencing holds significant promise in advancing precision medicine for ovarian cancer, tailoring treatments to individual tumor characteristics. However, further research and validation are needed before its widespread clinical implementation.

## 9 Conclusion & future perspectives

The field of single-cell sequencing is developing quickly whereby clinically relevant information such as intra-tumor heterogeneity, resistance towards treatment and tumor evolution can be collected by profiling single cells from cancer patients. Numerous fundamental objectives of cancer precision medicine (including prediction of treatment response, prognostication, and detection of treatment resistance) are possible to address at a higher resolution with scRNA-seq methods compared with traditional bulk average molecular phenotyping. Therefore, single-cell molecular phenotyping will overtake bulk average profiling in many application areas in the future. The way forward steps in the application of single-cell methods in the study of human cancers is to initiate studies that include larger patient cohorts, larger numbers of single cells and clinical outcomes.

## Author contributions

DB: Writing – review & editing, Writing – original draft, Methodology, Data curation, Conceptualization. NK: Writing – review & editing, Supervision. MP: Writing – review & editing, Supervision. NA: Writing – review & editing, Supervision, Resources, Project administration, Funding acquisition.
